# The influence of conjugated linoleic acid on the expression of peroxisome proliferator-activated receptor-γ and selected apoptotic genes in non-small cell lung cancer

**DOI:** 10.1007/s11010-020-03689-8

**Published:** 2020-01-29

**Authors:** Bartosz Kazimierz Słowikowski, Hanna Drzewiecka, Michał Malesza, Ida Mądry, Karolina Sterzyńska, Paweł Piotr Jagodziński

**Affiliations:** 1 Department of Biochemistry and Molecular Biology, Poznan University of Medical Sciences, Święcickiego 6 Street, 60-781 Poznan, Poland; 2 Department of Histology and Embryology, Poznan University of Medical Sciences, Święcickiego 6 Street, 60-781 Poznan, Poland

**Keywords:** Ppar-gamma, Non-small cell lung cancer, CLA, Apoptosis

## Abstract

In recent years, peroxisome proliferator-activated receptor-γ (PPARγ) has been intensively studied. Because its activation is often associated with changes in the expression level of various apoptotic genes, many studies have emphasized the role of PPARγ as an important anticancer agent. However, in different types of cancer, different genes are influenced by PPARγ action. Previous studies showed that conjugated linoleic acid (CLA) was able to induce apoptosis, upregulate *PPARG* gene expression and activate PPARγ protein in certain human cancer cell lines. Moreover, some PPARγ agonists inhibited the growth of human lung cancer cells through the induction of apoptosis. Nevertheless, the impact of CLA on PPARγ mRNA and protein levels in non-small cell lung cancer (NSCLC) cell lines has not been investigated thus far. Therefore, in our study, we analysed the influence of the c9,t11 linoleic acid isomer on the expression of *PPARG* and other genes involved in the apoptotic response (*BCL-2, BAX,* and *CDKN1A*) in two NSCLC cell lines of different histological origin (A549 and Calu-1) and in normal human bronchial epithelial Beas-2B cells. Cells were treated with several doses of c9,t11 CLA, followed by RNA and protein isolation, cDNA synthesis, real-time quantitative PCR (RT-qPCR) and Western blot analysis. We showed that the investigated CLA isomer was able to enhance the expression of PPAR*γ* in the examined cell lines and alter the mRNA and protein levels of genes involved in apoptosis. Fluorescent staining and MMT assay revealed the antiproliferative potential of CLA as well as its ability to activate pathways that lead to cell death.

## Introduction

According to recent statistics, lung cancer (LC) is the most commonly diagnosed type of tumour (11.6% cases worldwide). However, its curability has not significantly improved, and it remains one of the most common causes of cancer death among men (22%) and women (13.8%) [1]. Approximately 85% of all diagnosed LC cases are non-small cell lung cancers (NSCLCs), which include several histopathological subtypes, mainly adenocarcinoma and squamous cell carcinoma [2].

The crucial risk factors for developing LC are mainly age and long-term exposure to tobacco smoke. Nevertheless, a significant increase in the incidence of non-smokers with LC has been observed in recent years. This phenomenon may be related to the continuously growing degree of air pollution [3]. For these reasons, and for a better understanding of the molecular development of LC, it seems sensible to seek new therapeutic and prophylactic factors that will contribute to the reduction of LC risk.

Conjugated linoleic acid (CLA) belongs to the family of essential fatty acids and naturally occurs as a mixture of many positional and geometric isomers, where *cis*-9*,trans*-11 (c9,t11) and *cis-*10*,trans*-12 (c10,t12) are the most biologically active structures [4, 5]. The primary sources of CLA are the meat of ruminants and dairy products, in which approximately 90% of all CLAs occur as the c9,t11 isomer [4, 6]. CLA was discovered by Pariza et al. (1987) during a search for carcinogenic factors present in fried beef [6]. Eventually, CLA was found to possess anticarcinogenic and antiproliferative properties. Its pro-apoptotic action, as well as its inhibitory impact on cell growth, was revealed several times by using colon adenocarcinoma, gastric, prostate and breast cancer cell lines [7-10]. In addition, studies using animal models established that CLA exerts an inhibitory effect on chemically induced gastric, skin and breast cancer [7]. Research introduced by Sato et al. showed that the supplementation of CLA (2 g/day) can lead to its increase in serum to the concentration level (36 µM/l) that is considered to exhibit antiproliferative activity against human cancer cells [11].

It is commonly known that malignant tumours, including NSCLC, are characterized by dysregulation of many signalling pathways and insensitivity to pro-apoptotic signals, which leads to unlimited proliferation [10, 12]. One of the mechanism responsible for the induction of apoptosis in cancer cells is the stimulation of peroxisome proliferator-activated receptor-γ (PPARγ) by several factors, i.e. prostaglandins, glitazones and fatty acids (including CLA, which is its natural ligand) [13, 14]. PPAR-γ is encoded by *PPARG* gene and belongs to the family of nuclear receptors that act as transcription factors. PPAR-γ regulates the expression of genes related to carbohydrate and lipid metabolism, immune system function, growth, differentiation and apoptosis [15]. PPAR-γ exerts its effect through two different mechanisms. First, as a ligand-dependent transcription factor, PPAR-γ can bind to DNA in the promoter region of genes with sequences known as peroxisome proliferator response elements (PPREs). Second, PPAR-γ can control gene expression independently of PPREs by associating with activator proteins 1 and 2, which act as known transcription factors [16, 17].

Usually, activation of PPAR-γ results in increased expression of genes that encode proteins responsible for the promotion of apoptosis (e.g. BAX, BAK, BAD, BID, and p21) and decreased expression of genes encoding anti-apoptotic agents (e.g. BCL-2) [16, 18]. This process results in enhanced programmed cell death, which limits the viability and proliferation of cancer cells [8-10, 17-19]. It was shown that stimulation of PPAR-γ in cancers affects the expression of several genes associated with apoptosis, i.e. *MYC* in thyroid cancer, *GADD153* (growth arrest and DNA damage-inducible 153) in colon cancer and LC and *POX* (proline oxidase) in colon cancer. Furthermore, activation of PPAR-γ inhibits the development of colon, lung, and breast cancer cells in vitro and exerts a suppressive impact on the progression of NSCLC in animal models [20, 21].

Intensive studies on CLA showed that its antiproliferative effect is a complex and multidirectional process. One of the antiproliferation mechanisms may be connected with the activation of PPAR-γ. In vitro research performed on hepatic cancer cell lines pinpointed CLA as an activation ligand of PPAR-γ as well as an enhancer of *PPARG* expression, suggesting its impact on pro-apoptotic actions in cancer cells [6, 8, 12, 18].

In contrast, in other cells (e.g. neurons and cardiac cells), PPAR-γ has protective effects. It was demonstrated that PPAR-γ upregulated BCL-2 and induced the stability of mitochondria, thus providing protection against oxidative stress and associated apoptosis [22, 23]. The mechanism of this specific phenomenon may be related to the concentration of the stimulating ligand—high levels of PPAR-γ ligands may have pro-apoptotic properties, while at lower concentrations, they may exert anti-apoptotic actions [24]. This particular curiosity is called "a U-shaped dose–response relationship" or "hormesis" and is widely documented, especially in the field of pharmacology and toxicology. In regard to concentration, some substances may act positively or negatively [25].

Because LC remains the most common cancer diagnosed, there is a need to look for new possible protective factors. CLA, which is present in various types of food and very commonly used in dietary supplements, may be one such factor. The main aim of our study was to investigate the influence of the most common c9, t11 CLA isomer on the expression of *PPARG* and selected pro- and anti-apoptotic genes (*BAX*, *CDKN1A*, and *BCL-2*) as well as its impact on LC cells proliferation and viability. Experiments were performed by using two different NSCLC cell lines (A549 and Calu-1) and normal human bronchial epithelial cells (Beas-2B).

## Material and methods

### Cell culture

Human A549 (adenocarcinoma) and Calu-1 (squamous cell carcinoma) NSCLC cells were purchased from American Type Culture Collection (Rockville, MD). Normal human bronchial epithelial Beas-2B cells were kindly provided by Dr M. Rusin from the Maria Skłodowska-Curie Memorial Cancer Center and Institute of Oncology, Gliwice Branch, Poland. A549 and Calu-1 cell lines were routinely maintained in RPMI 1640 medium (Sigma Aldrich, St. Louis, MO), while Beas-2B cells were cultured in DMEM/F12 medium (Sigma Aldrich, St. Louis, MO). Both media were supplemented with 10% heat-inactivated foetal bovine serum, 2 mM glutamine, and 1% penicillin–streptomycin solution (10.000 units of penicillin and 10 mg of streptomycin/ml; Sigma Aldrich, St. Louis, MO). Cells were grown at 37 °C in humidified air with 5% CO_2_.

### Incubation with c9, t11 CLA

Because we expected an intensified apoptotic effect, we carried out several minor experiments to establish appropriate doses of CLA and incubation times for each cell line (in order to avoid cytotoxicity and misleading results). Our assessment was based mainly on the measurement of cell number and viability in the subsequent days of incubation. This process was crucial for an efficient protein and RNA isolation, as well as for cDNA synthesis. For this purpose, we used Trypan blue staining (Thermo Fisher, Waltham, USA) and EVE™ Automated Cell Counter (NanoEnTek, Seoul, North Korea). In order to investigate the effect of c9, t11 CLA (Sigma Aldrich, St. Louis, MO) on *PPARG, BCL-2,BAX,, and CDKN1A* expression level we found the following conditions to be the most suitable:

**A549** cells were cultured for 24, 48 and 72 h in the presence of three different doses of c9, t11 CLA (50 µM, 100 µM, and 200 µM).

**Calu-1** cells were grown for 24 and 48 h using three concentrations of c9, t11 CLA (25 µM, 50 µM, and 75 µM).

**Beas-2B** cells were incubated for 24, 48 and 72 h with c9, t11 CLA at concentrations of 25 µM, 50 µM, and 75 µM.

The stock solutions of c9, t11 CLA were prepared in DMSO, aliquoted and stored at − 20 °C until later use. Before each experiment, c9, t11 CLA from the stock solution was diluted in cell culture media to the desired concentration and added to culture vessels. The stimulation media were exchanged every 24 h. All experiments were performed in three biological repeats and included a control sample treated with appropriate amounts of DMSO (vehicle control), the concentration of which never exceeded 0.1%, which is standard culturing practice.

### RNA isolation, reverse transcription, and Real-time quantitative PCR

Total cellular RNA was isolated by TRIzol® (Thermo Fisher, Waltham, USA) according to the manufacturer's protocol. The quantity and purity of the obtained material was evaluated spectrophotometrically by using a NanoDrop™ One (Thermo Fisher, Waltham, USA). To determine the integrity of isolated RNA, we performed agarose gel electrophoresis. Isolated samples were stored at 80 °C until further analysis.

To obtain high-quality cDNA, we used SuperScript IV reverse transcriptase (Thermo Fisher Scientific, Waltham, USA) along with the mixture of oligo dT primers and hexamers (according to the manufacturer's protocol). Real-time quantitative PCR (RT-qPCR) was carried out in the Light Cycler®480 Real-Time PCR System (Roche Diagnostics GmbH, Mannheim, Germany). *PPARG, BCL-2,* and *CDKN1A* genes were analysed using LightCycler® 480 SYBR Green I Master (Roche Diagnostics GmbH, Mannheim, Germany). Because the *BAX* sequence is very repetitive, to preserve specificity, we used a pre-designed PrimePCR™ Probe Assay (Bio-Rad, Hercules, California, USA) to estimate the *BAX* expression level.

The efficiency of all reactions was calculated by generating standard curves from a serial dilution of cDNA template mix from all of the samples. Every experiment included negative, no-template, non-transcribed RNA and genomic DNA controls. For calibration purposes, we used 1 µl of cDNA template mix. The quantity of examined transcripts in each analysis was standardized with the geometric mean of three housekeeping genes—porphobilinogen deaminase (*PBGD*); human mitochondrial ribosomal protein L19 (*hMRPL19*); and RNA polymerase II subunit A (*POLR2A*). The melting curve analysis and agarose gel electrophoresis were performed to confirm the specificity of the SYBR Green PCR products.

### Protein isolation, sodium dodecyl sulphate–polyacrylamide gel electrophoresis (SDS–PAGE) and Western blotting analysis

For protein isolation, harvested cells were mixed with RIPA lysis buffer (Sigma Aldrich, St. Louis, USA) enriched with Roche cOmplete™ protease inhibitor cocktail (Sigma Aldrich, St. Louis, USA), incubated on ice for 30 min and centrifuged at 10,000×*g* for 10 min at 4 °C in order to remove cellular debris. Next, 30 μg of total protein was resuspended in sample loading buffer, incubated in 99C for 12 min, cooled down on ice and separated on 12% Tris–Glycine gels, using sodium dodecyl sulphate–polyacrylamide gel electrophoresis (SDS-PAGE). Proteins from gels were electrotransfered to a nitrocellulose membrane, which was then blocked with 5% non-fat dry milk in 1 × concentrated Tris–HCl saline/Tween buffer. After blocking, membranes were incubated in 4˚C with anti-PPAR-γ or anti-BCL-2 monoclonal antibody (Santa-Cruz, California, USA) at the dilution of 1:500. This process was followed by washing membranes in 1 × Tris–HCL saline/Tween buffer and placing them in the solution of secondary antibody conjugated with horseradish peroxidase (1:1000); (Santa-Cruz, California, USA). Immunochemiluminescent signal was revealed using SuperSignal West Femto Chemiluminescent Substrate (Thermo Fisher, Waltham, USA) and ChemiDoc MP imaging system (Bio-Rad, Hercules, California, USA). Next, membranes were restriped and incubated with anti-GAPDH anibody (FL-335) (1:5000) (Santa-Cruz, California, USA) followed by incubation with secondary goat anti-rabbit HRP-conjugated antibody (1:5000). The amount of analysed proteins was demonstrated as the investigated protein-to-GAPDH optical density ratio. Optical density was measured by using ImageJ2x programme [26].

### MTT cell proliferation assay

To determine the cytotoxic and antiproliferative effect of *c9, t11* CLA on examined cell lines we used MTT proliferation assay (Sigma Aldrich, St. Louis, USA). Cells were cultured in DMEM/F12 (Beas-2B) or RPMI 1640 (A549, Calu-1) medium enriched with 10% FBS for 24 h and then seeded into 96-well plates at the density of 3.5 × 10^3^ (A549 and Beas-2B) and 7.0 × 10^3^ (Calu-1) cells per well. Next, cells were incubated in the presence of different doses of CLA (25 μM, 50 μM, 75 μM for Beas-2B and Calu-1; 50 μM, 100 μM, 200 μM for A549) for 24, 48 and 72 h. After the incubation period, all media were removed and replaced by 200 μl of 10% MTT solution (5 mg of MTT substrate per 1 ml of PBS) in FBS-free cell culture medium and left for 4 h in the incubator. After this time MTT mixture was carefully discarded and newly formatted formazan crystals were dissolved by adding 50 μl of DMSO into each well. The absorbance was measured by Epoch Plate Spectrophotometer (BioTek, Vinooski,VT, USA). Each experiment was performed in 8 biological repeats for each CLA concentration and included 8 vehicle controls (with DMSO).

### Fluorescence microscopy

To evaluate the impact of *c9, t11* CLA on cell cell membrane integrity we performed bis-benzimide (Hoechst 33,342; Sigma Aldrich, St. Louis, USA) and propidium iodide (PI); (Sigma Aldrich, St. Louis, USA) double fluorescent staining. Hoechst 33,342 (blue) has an ability to penetrate the intact cell membranes while the PI (red), due to its electrical charge is a membrane impermeant dye. However, PI stains cells with damaged membranes, which are characteristic for late-apoptotic and dead cells. Before staining, cells were incubated in the presence or absence (vehicle controls) of different doses of CLA (as described above) for 72 h in chamber slides (Thermo Fisher, Waltham, USA). After the experiment, cells were washed twice with 1 × PBSand incubated with Hoechst 33,342 solution (1 μg/ml) for 10 min in the dark at room temperature. Next, cells were washed again (3 × with 1 × PBS), and incubated with PI solution (1 μg/ml) for 5 min and rinsed 3 times with PBS before visualization. The fluorescent signal was detected by Zeiss Axio-Imager Z1 (Carl Zeiss, Microscopy GmbH, Oberkochen, Germany).

### Statistical analysis

The normality of the observed data distribution was established by the Shapiro–Wilk test. The one-way analysis of variance (ANOVA) with post-hoc HSD Tukey–Kramer test were used to compare the mean values of *PPARG, BCL-2, BAX,* and *CDKN1A* transcript levels and to identify statistically significant differences between the control samples and the c9,t11 CLA-treated cells. The ANOVA with post-hoc HSD Tukey–Kramer test was used to compare mean values of the absorbancy level of MTT assay. Gene expression as well as protein level is displayed as the multiplicity of the respective controls at selected incubation times. To determine whether there are any significant associations between the expression levels of investigated genes, we performed a Pearson correlation analysis (the distribution of the data was normal). A *p* value < 0.05 was considered statistically significant. All statistical analyses were performed by using GraphPad in Stat 3.10 and STATISTICA 13 software.

## Results

### Evaluation of the c9,t11 CLA effect on the mRNA and protein levels of the investigated genes in A549 cell line

**Fig. 1:**
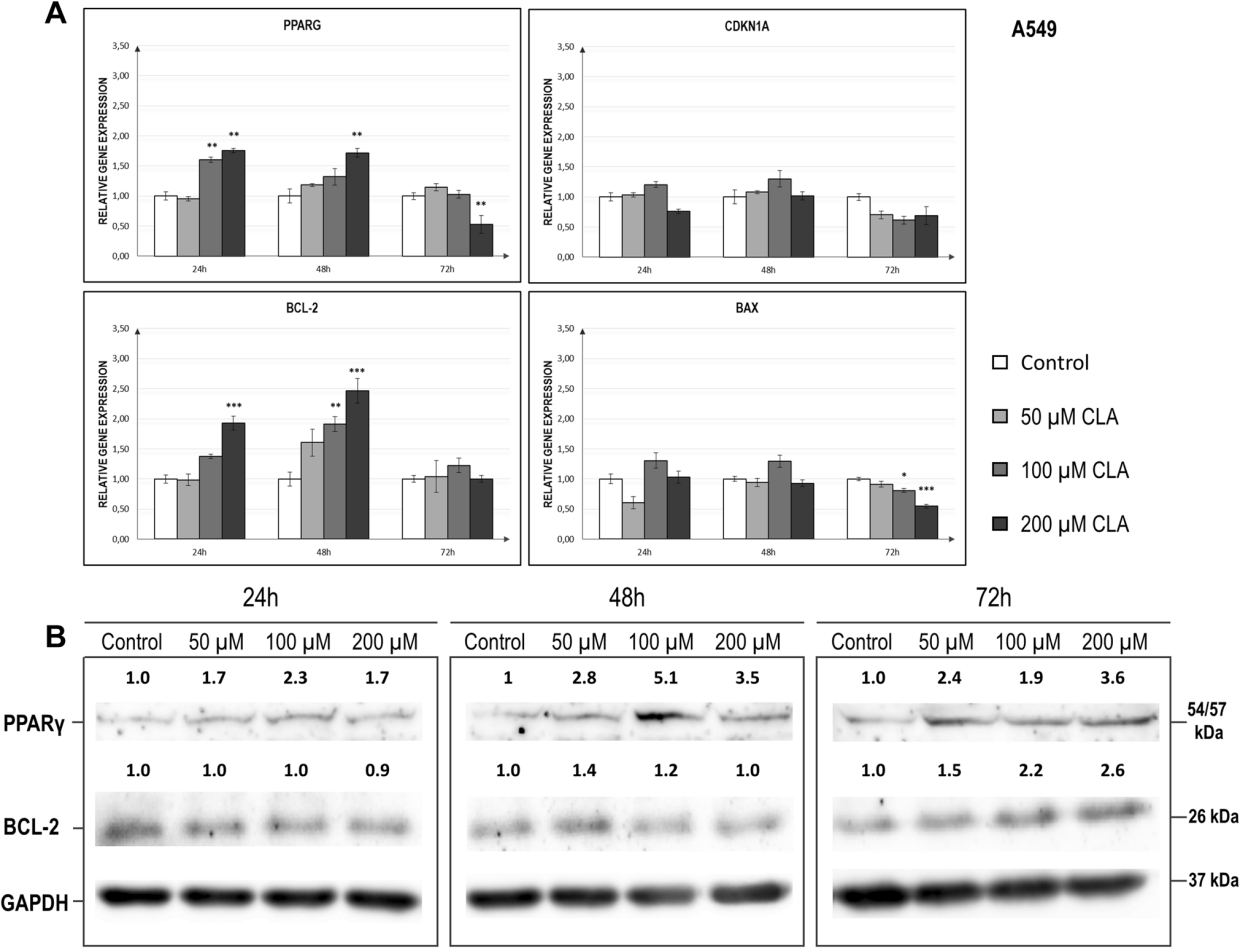
**a** Relative expression levels of *PPARG, CDKN1A, BCL-2* and *BAX* genes in A549 cell line, after treatment with different doses of c9, t11 CLA in selected incubation times. Cells were maintained for 24 h to 72 h in the absence (controls, only DMSO) or in the presence of c9, t11 CLA at concentrations of 50, 100 and 200 µM. After incubation, cells were used for total RNA isolation and RT-qPCR analysis. Each gene transcript level is displayed as the mean multiplicity of the respective control samples ± SEM. Statistically significant differences between control and tested cells are marked with asterisks (*p value < 0.05; **p value < 0.01; and ***p value < 0.001). **b** Representative image of Western Blot analysis of PPAR-γ and BCL-2 status in the A549 cell line. Cells were maintained for 24 h to 72 h in the absence (controls, only DMSO) or in the presence of c9, t11 CLA at concentrations of 50, 100 and 200 µM. Proteins were isolated by using RIPA buffer, separated by 10% SDS-PAGE and detected by Western Blotting. The numbers above the images of protein bands are optical density ratio values of PPAR-γ or BCL-2 to GAPDH for each sample. The ratio of PPAR-γ or BCL-2 to GAPDH for control cells in each incubation time was assumed to be 1. The protein level was displayed as the mean multiplicity of the respective control sample

Data concerning the relative expression levels of the investigated genes and Western blot results for A549 cells are presented in Fig. 1 and Table 1. Our analysis revealed that *PPARG* transcript levels significantly increased after 24 h (*p* = 0.0006) and 48 h (*p* = 0.0125) of c9,t11 *CLA* stimulation (Fig. 1a). However, after 72 h of incubation with CLA at a dose of 200 µM, we noticed a drop in the *PPARG* expression level (*p* = 0.0077). Statistically significant difference in *PPARG* mRNA levels at 72 h occurred between untreated vehicle controls and the highest concentration of investigated compound (*p* < 0.01). According to the chart (Fig. 1a), we observed the following pattern: the higher the concentration of stimulant was, the greater the increase after 24 and 48 h and the greater the decrease in expression of *PPARG* after 72 h. The increase in transcript levels of *PPARG* was confirmed by changes in protein amounts (Fig. 1b). A similar trend was noticed in t case of t *BCL-2* gene (Fig. 1a). CLA-treated cells demonstrated meaningful upregulation of *BCL-2* expression after 24 h (*p* = 0.001) and 48 h (*p* = 0.0019) which returned to the basal level after 72 h. The protein level of BCL-2 has also increased, although this effect was mostly observed after 72 h of the CLA treatment (Fig. 1b).The other investigated genes revealed only subtle changes in the expression level. The* BAX* transcripts level decreased after 72 h of c9,t11 CLA treatment at highest concentration-doses—(100 and 200 µM); (*p* = 0.0001); (Fig. 1b). The same trend was visible for *CDKN1A,* but the data did not reach statistical significance (*p* = 0.0596); (Fig. 1a). Moreover, Pearson correlation analysis revealed a strong correlation between *PPARG* and *BCL-2* expression levels in A549 cells (*p* = 0.0001; *r* = 0.5922; *r*^2^ = 0.3507).

**Table 1:** Statistical analysis of *PPARG, BCL-2, BAX* and *CDKN1A* expression level in control and c9, t11 CLA-treated A549 cells in different times of incubation; *p* value < 0.05 was considered statistically significant

Cell line/gene		A549				
	Time of incubation	Relative normalized expression				
		Control (± SEM)	50 uM (± SEM)	100 uM (± SEM)	200 uM (± SEM)	ANOVA *p* value
*PPARG*	24 h	1.00	0.95	1.60^**^	1.76^**^	**0.0006**
		(± 0.05)	(± 0.02)	(± 0.12)	(± 0.14)	
	48 h	1.00	1.18	1.32	1.72^**^	**0.0125**
		(± 0.03)	(± 0.08)	(± 0.10)	(± 0.19)	
	72 h	1.00	1.15	1.03	0.53^**^	**0.0077**
		(± 0.04)	(± 0.05)	(± 0.14)	(± 0.11)	
*BCL-2*	24 h	1.00	0.99	1.37	1.93***	**0.0001**
		(± 0.07)	(± 0.10)	(± 0.04)	(± 0.11)	
	48 h	1.00	1.61	1.91^*^	2.47^**^	**0.0019**
		(± 0.11)	(± 0.22)	(± 0.12)	(± 0.20)	
	72 h	1.00	1.04	1.23	1.00	> 0.05
		(± 0.05)	(± 0.26)	(± 0.12)	(± 0.15)	
*BAX*	24 h	1.00	0.76	1.30	1.03	> 0.05
		(± 0.04)	(± 0.10)	(± 0.13)	(± 0.10)	
	48 h	1.00	0.94	1.29	0.93	> 0.05
		(± 0.04)	(± 0.07)	(± 0.10)	(± 0.06)	
	72 h	1.00	0.91	0.81^*^	0.55^***^	**0.0001**
		(± 0.03)	(± 0.05)	(± 0.03)	(± 0.03)	
*CDKN1A*	24 h	1.00	1.03	1.11	0.98	> 0.05
		(± 0.04	(± 0.03)	(± 0.05)	(± 0.04)	
	48 h	1.00	1.08	1.30	1.02	> 0.05
		(± 0.02)	(± 0.02)	(± 0.14)	(± 0.07)	
	72 h	1.00	0.70	0.61	0.69	> 0.05
		(± 0.05)	(± 0.06)	(± 0.06)	(± 0.15)	

Asterisk marked results significantly differs compared to untreated control—samples measurements (*p value < 0.05; **p value < 0.01; ***p value < 0.001)

### Evaluation of the c9, t11 CLA effect on the mRNA and protein levels of the investigated genes in Calu-1 cell line

**Fig. 2:**
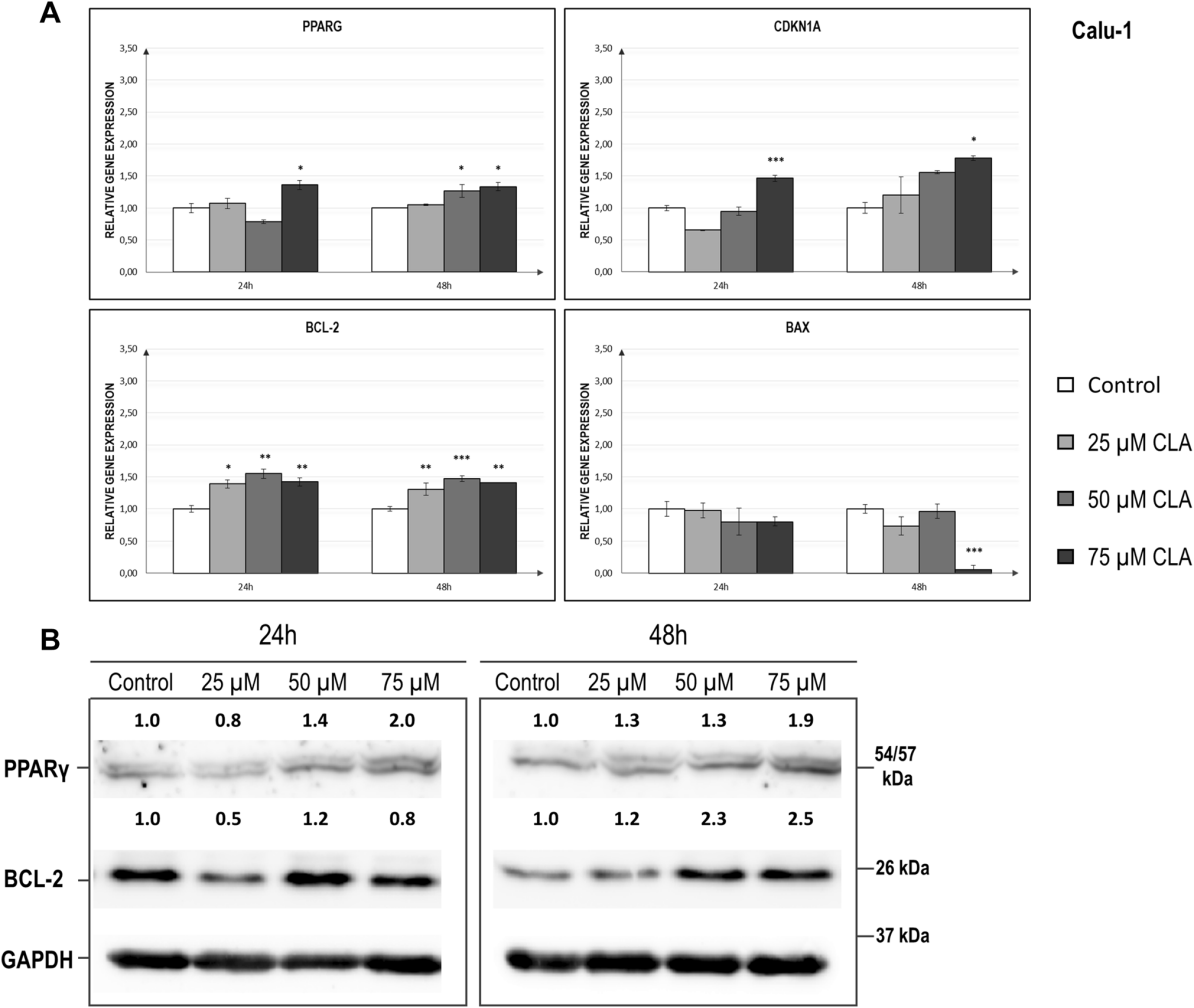
**a** Relative expression levels of *PPARG*, *CDKN1A, BCL-2* and *BAX* genes in the Calu-1 cell line, after treatment with different doses of c9, t11 CLA in selected incubation times. Cells were maintained for 24 h to 48 h in the absence (controls) or in the presence of c9, t11 CLA at concentrations of 25, 50 and 75 µM. After incubation, cells were used for total RNA isolation and RT-qPCR analysis. Each gene transcript level is displayed as the mean multiplicity of the respective control samples ± SEM. Statistically significant differences between control and tested samples are marked with asterisks (*p value < 0.05; **p value < 0.01; and ***p value < 0.001). **b** Representative image of Western Blot analysis of PPAR-γ and BCL-2 status in Calu-1 cell line. Cells were maintained for 24 h to 72 h in the absence (controls, only DMSO) or in the presence of c9, t11 CLA at concentrations of 25, 50 and 75 µM. Proteins were isolated by using RIPA buffer, separated by 10% SDS-PAGE and detected by Western Blotting. The numbers above the images of protein bands are optical density ratio values of PPAR-γ or BCL-2 to GAPDH for each sample. The ratio of PPAR-γ or BCL-2 to GAPDH for control cells in each incubation time was assumed to be 1. The protein level was displayed as the mean multiplicity of the respective control sample

Data demonstrating changes in the relative expression levels of the investigated genes in Calu-1 cells are presented in Fig. 2 and Table 2. Our analysis showed that the changes in the transcript levels of the examined genes in the Calu-1 cell line were not as striking as in A549 cells, although we noticed some significant alterations. *PPARG* and *BCL-2* mRNA levels were elevated after 24 h (*p* = 0.0019 and *p* = 0.0031, respectively) and 48 h (*p* = 0.01 and *p* = 0.0010, respectively), especially in the presence of the highest dose of c9,t11 CLA (75 µM); (Fig. 2a). Although the differences in transcript levels were not so prominent, the effect of CLA was more visible in case of proteins content. We observed elevated amounts of PPAR-γ in the CLA-treated cells, especially at higher doses of the compound (Fig. 2b). Our Western blot analysis also demonstrated an increased BCL-2 protein values after 48 h of incubation period. Moreover, we showed that a statistically significant correlation occurred between *PPARG* and *BCL-2* transcript levels (*p* = 0.04, *r* = 0.37, *r*^2^ = 0.13). However, the correlation was much weaker compared to the correlation between these two genes in the A549 cell line. Next, a small but significant upregulation of *CDKN1A* expression was observed when CLA was used at the dose of 75 µM, regardless of time (for 24 h *p* = 0.001 and for 48 h *p* = 0.0221); (Fig. 2a). *p* = *p* = Similar to the results in A549 cells, our study also revealed a substantial drop in *BAX* expression level after 48 h of incubation with the highest applied dose of CLA (Fig. 2a).

**Table 2:** Statistical analysis of *PPARG, BCL-2, BAX* and *CDKN1A* expression level in control and c9,t11 CLA-treated Calu-1 cells in different times of incubation; *p* value < 0.05 was considered statistically significant

Cell line/gene		Calu-1				
	Time of incubation	Relative normalized expression				
		Control (± SEM)	25 uM (± SEM)	50 uM (± SEM)	75 uM (± SEM)	ANOVA *p* value
*PPARG*	24 h	1.00	1.07	0.84	1.36^*^	**0.0019**
		(± 0.07)	(± 0.08)	(± 0.03)	(± 0.07)	
	48 h	1.00	1.05	1.27^*^	1.33^*^	**0.01**
		(± 0.01)	(± 0.01)	(± 0.10)	(± 0.06)	
*BCL-2*	24 h	1.00	1.31^*^	1.47^**^	1.42^**^	**0.0031**
		(± 0.05)	(± 0.07)	(± 0.07)	(± 0.06)	
	48 h	1.00	1.39^**^	1.55^***^	1.41^**^	**0.0010**
		(± 0.04)	(± 0.10)	(± 0.05)	(± 0.00)	
*BAX*	24 h	1.00	0.97	0.80	0.80	> 0.5
		(± 0.12)	(± 0.12)	(± 0.21)	(± 0.15)	
	48 h	1.00	0.74	0.96	0.06^***^	**0.00040**
		(± 0.07)	(± 0.14)	(± 0.11)	(± 0.00)	
*CDKN1A*	24 h	1.00	0.84	0.95	1.46^***^	**0.001**
		(± 0.04)	(± 0.01)	(± 0.07)	(± 0.05)	
	48 h	1.00	1.20	1.56	1.78^*^	**0.0221**
		(± 0.08)	(± 0.28)	(± 0.03)	(± 0.03)	

Asterisk marked results significantly differs compared to untreated control—samples measurements (*p value < 0.05; **p value < 0.01; ***p value < 0.001)

### Evaluation of the c9, t11 CLA effect on the mRNA and protein levels of the investigated genes in Beas-2B cell line

**Fig. 3:**
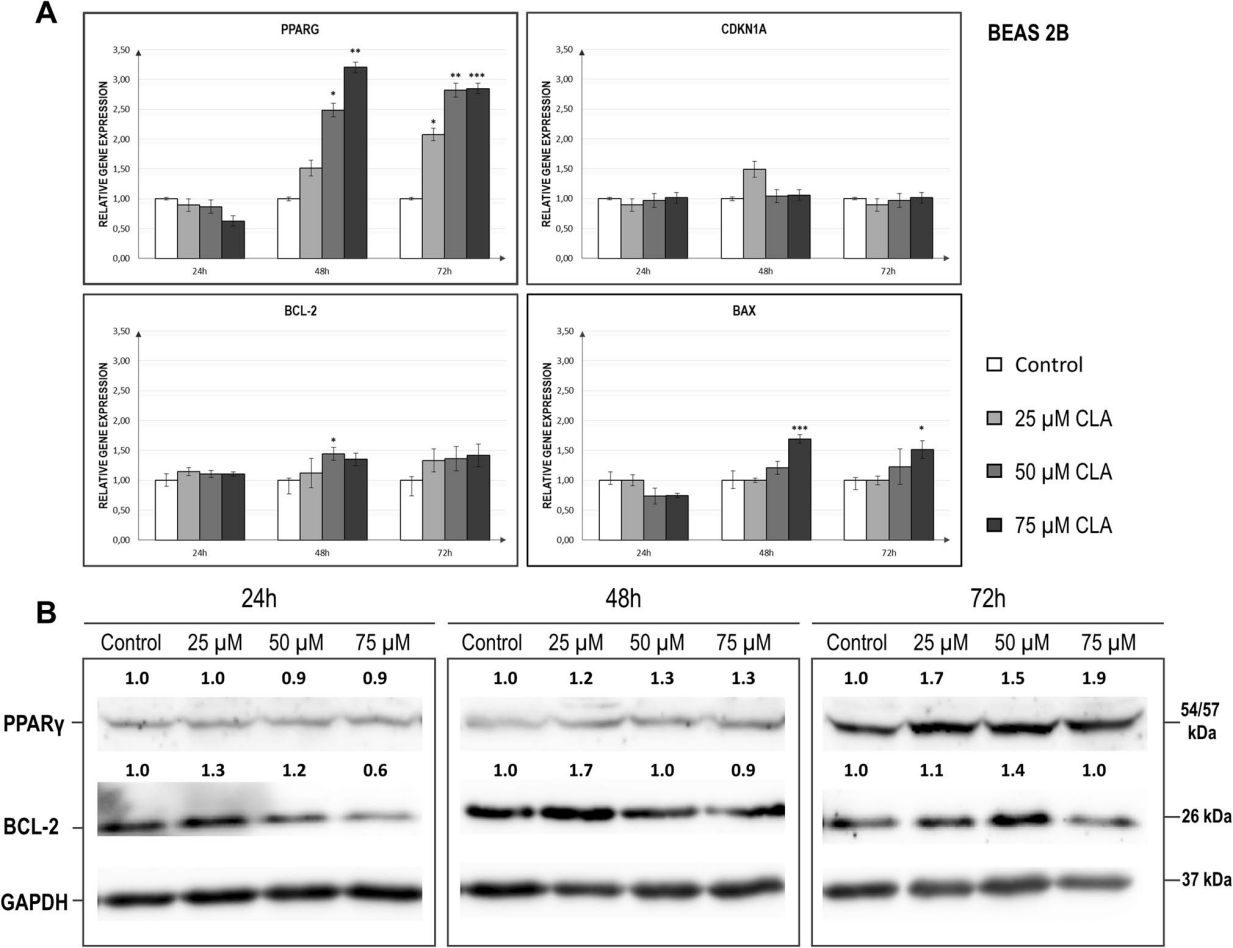
**a** Relative expression levels of *PPARG, CDKN1A, BCL-2* and *BAX* genes in the Beas-2B cell line, after treatment with different doses of c9, t11 CLA in selected incubation times. Cells were maintained for 24 h to 72 h in the absence (controls) or in the presence of c9, t11 CLA at concentrations of 25, 50 and 75 μM. After incubation, cells were used for total RNA isolation and RT-qPCR analysis. Each gene transcript level is displayed as the mean multiplicity of the respective control samples ± SEM. Statistically significant differences between control and tested samples are marked with asterisks (*p value < 0.05; **p value < 0.01; and ***p value < 0.001). **b** Representative image of Western Blot analysis of PPAR-γ and BCL-2 status in Beas-2B cell line. Cells were maintained for 24 h to 72 h in the absence (controls, only DMSO) or in the presence of c9, t11 CLA at concentrations of 25, 50 and 75 µM. Proteins were isolated by using RIPA buffer, separated by 10% SDS-PAGE and detected by Western Blotting. The numbers above the images of protein bands are optical density ratio values of PPAR-γ or BCL-2 to GAPDH for each sample. The ratio of PPAR-γ or BCL-2 to GAPDH for control cells in each incubation time was assumed to be 1. The protein level was displayed as the mean multiplicity of the respective control sample

All results concerning the impact of the c9,t11 CLA isomer on the expression level of analysed genes are shown in Fig. 3 and Table 3. During the analysis of Beas-2B samples, we observed the most noticeable and substantial increase of *PPARG* transcript levels in stimulated cells compared to the other stimulated NSCLC cell lines (Fig. 3a). *PPARG* overexpression was noticed after 48 h (*p* = 0.0016) and 72 h (*p* = 0.0015) of incubation, regardless of the concentration of the stimulant. This effect was confirmed by the protein analysis, however the most visible changes were revealed after 72 h of treatment (Fig. 3b). Furthermore, the application of c9,t11 CLA at a dose of 75 µM resulted in an increase in the quantity of *BAX* mRNA level after 48 h (*p* = 0.0016) and 72 h (*p* = 0.0103) of treatment (Fig. 3a). Interestingly, in the contrary to previous results from NSCLC cell lines, we found a strong positive correlation between *PPARG* and *BAX* transcript levels (*p* = 0.0001; *r* = 0.6399; *r*^2^ = 0.4094) in Beas-2B cells. The other investigated genes did not reveal major changes. Although we also observed a certain trend towards elevated *BCL-2* expression, this outcome did not reach statistical significance (Fig. 3a). Lastly, the level of *CDKN1A* was not affected by c9,t11 CLA (Fig. 3a).

**Table 3:** Statistical analysis of *PPARG, BCL-2, BAX* and *CDKN1A* expression level in control and c9, t11 CLA-treated Beas-2B cells in different times of incubation; *p* value < 0.05 was considered statistically significant

Cell line/gene		Beas-2b				
	Time of incubation	Relative normalized expression				
		Control (± SEM)	25 uM (± SEM)	50 uM (± SEM)	75 uM (± SEM)	ANOVA *p* value
*PPARG*	24 h	1.00	0.89	0.87	0.63	> 0.5
		(± 0.06)	(± 0.22)	(± 0.14)	(± 0.05)	
	48 h	1.00	1.52	2.49^*^	3.21^**^	**0.0016**
		(± 0.11)	(± 0.23	(± 0.41)	(± 0.23)	
	72 h	1.00	2.08^*^	2.82^**^	2.85^***^	**0.0015**
		(± 0.09)	(± 0.39)	(± 0.22)	(± 0.09)	
*BCL-2*	24 h	1.00	1.14	1.11	1.11	> 0.5
		(± 0.11)	(± 0.07)	(± 0.06)	(± 0.04)	
	48 h	1.00	1.12	1.44^*^	1.35	**0.0195**
		(± 0.04)	(± 0.24)	(± 0.11)	(± 0.10)	
	72 h	1.00	1.33	1.36	1.42	> 0.5
		(± 0.06)	(± 0.19)	(± 0.20)	(± 0.19)	
*BAX*	24 h	1.00	0.79	0.74	0.74	> 0.5
		(± 0.14)	(± 0.09)	(± 0.13)	(± 0.04)	
	48 h	1.00	0.75	1.21	1.69^***^	**0.0001**
		(± 0.16)	(± 0.04	(± 0.11	(± 0.07)	
	72 h	1.00	0.90	1.23	1.51^*^	**0.0103**
		(± 0.05)	(± 0.07)	(± 0.30)	(± 0.15)	
*CDKN1A*	24 h	1.00	0.90	0.97	1.01	> 0.5
		(± 0.02)	(± 0.11)	(± 0.12	(± 0.09)	
	48 h	1.00	1.20	1.04	1.06	> 0.5
		(± 0.03)	(± 0.13)	(± 0.11)	(± 0.09)	
	72 h	1.00	0.90	0.97	1.01	> 0.5
		(± 0.02)	(± 0.11)	(± 0.12)	(± 0.09)	

Asterisk marked results significantly differs compared to untreated control—samples measurements (*p value < 0.05; **p value < 0.01; ***p value < 0.001)

### Evaluation of the c9,t11 CLA impact on cell viability, proliferation and membrane integrity

**Fig. 4:**
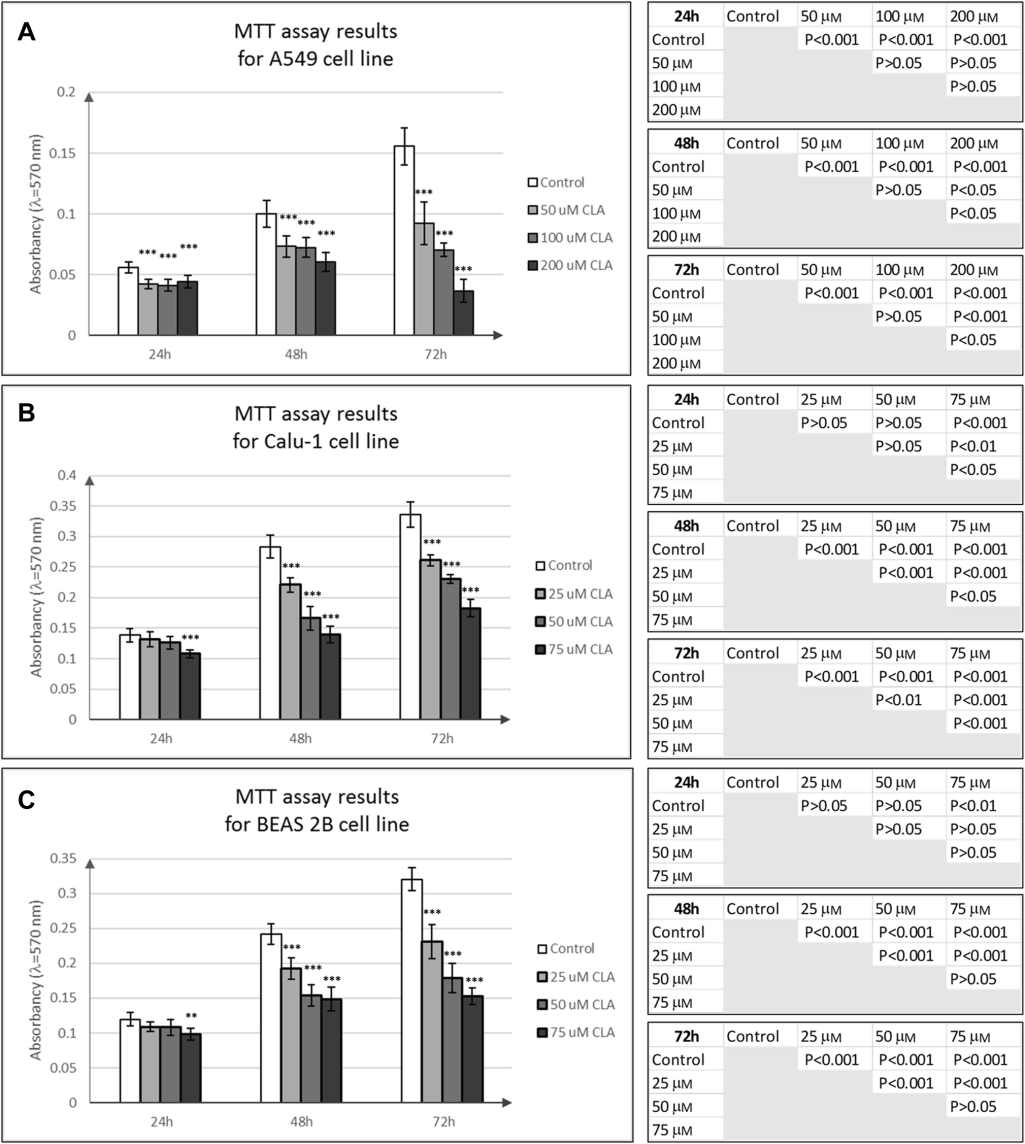
Effect of different doses of CLA on the proliferation of A549 (**a**), Calu-1 (**b**) and Beas-2B (**c**) cells. Statistically significant differences between control and tested samples are marked with asterisks (*p value < 0.05; **p value < 0.01; and ***p value < 0.001)

**Fig. 5:**
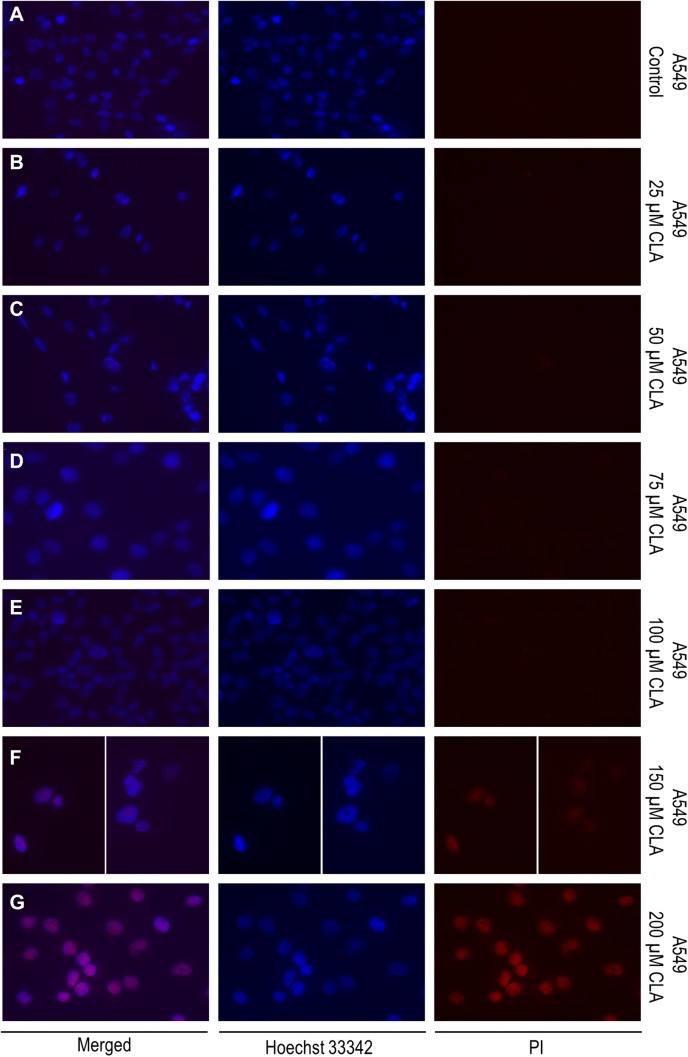
Double, fluorescent staining of A549 cells with Hoechst 33,342 (1 μg/ml) and PI (1 μg/ml)—**a** control (CLA-untreated, DMSO-treated cells); **b** cells treated with 25 μM CLA; **c** cells treated with 50 μM CLA; **d** cells treated with 75 μM CLA; **e** cells treated with 100 μM CLA; **f** cells treated with 150 μM CLA; **g** cells treated with 200 μM CLA. Fluorescent signal was detected by fluorescence microscope magnification =  × 40

**Fig. 6:**
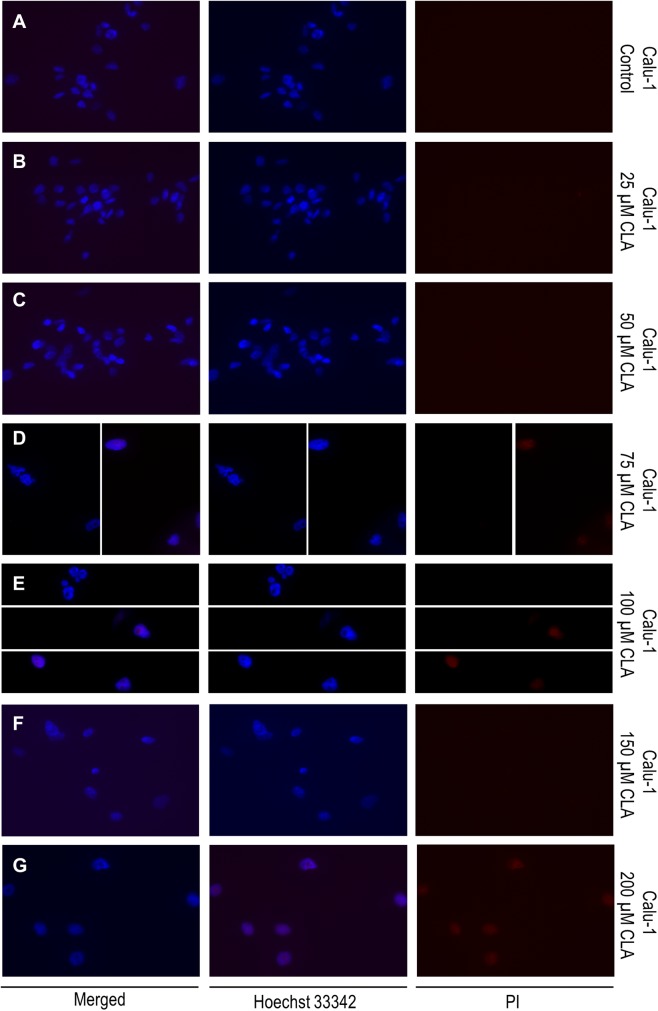
Double, fluorescent staining of Calu-1 cells with Hoechst 33,342 (1 μg/ml) and PI (1 μg/ml)—**a** control (CLA-untreated, DMSO-treated cells); **b** cells treated with 25 μM CLA; **c** cells treated with 50 μM CLA; **d** cells treated with 75 μM CLA; **e** cells treated with 100 μM CLA; **f** cells treated with 150 μM CLA; **g** cells treated with 200 μM CLA. Fluorescent signal was detected by fluorescence microscope magnification =  × 40

**Fig. 7:**
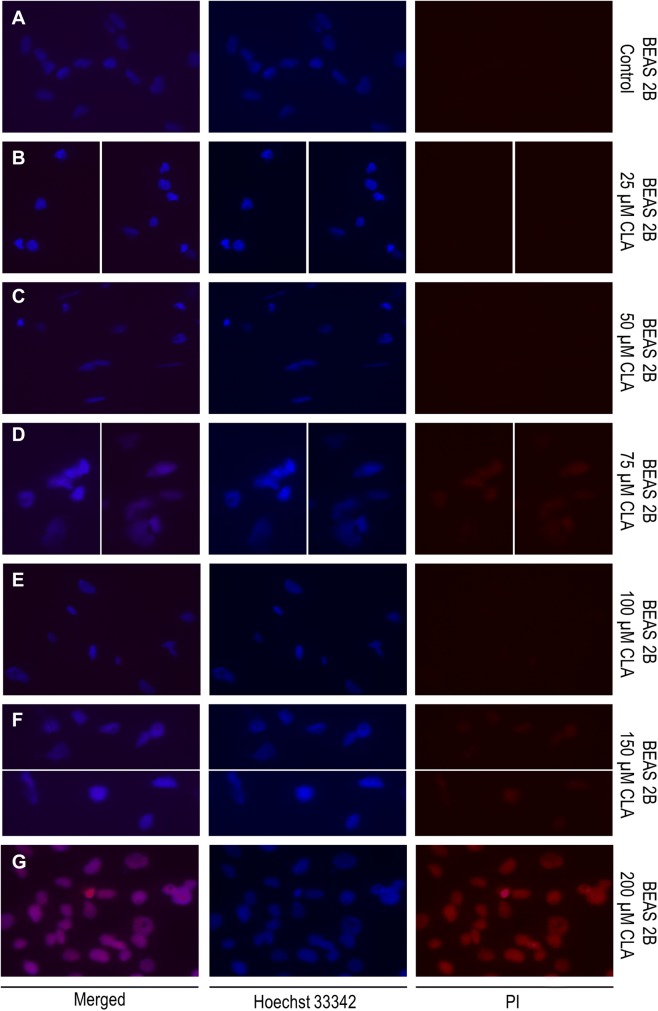
Double, fluorescent staining of Beas-2B cells with Hoechst 33,342 (1 μg/ml) and PI (1 μg/ml)—**a** control (CLA-untreated, DMSO-treated cells); **b** cells treated with 25 μM CLA; **c** cells treated with 50 μM CLA; **d** cells treated with 75 μM CLA; **e** cells treated with 100 μM CLA; **f** cells treated with 150 μM CLA; **g** cells treated with 200 μM CLA. Fluorescent signal was detected by fluorescence microscope magnification =  × 40

**Fig. 8:**
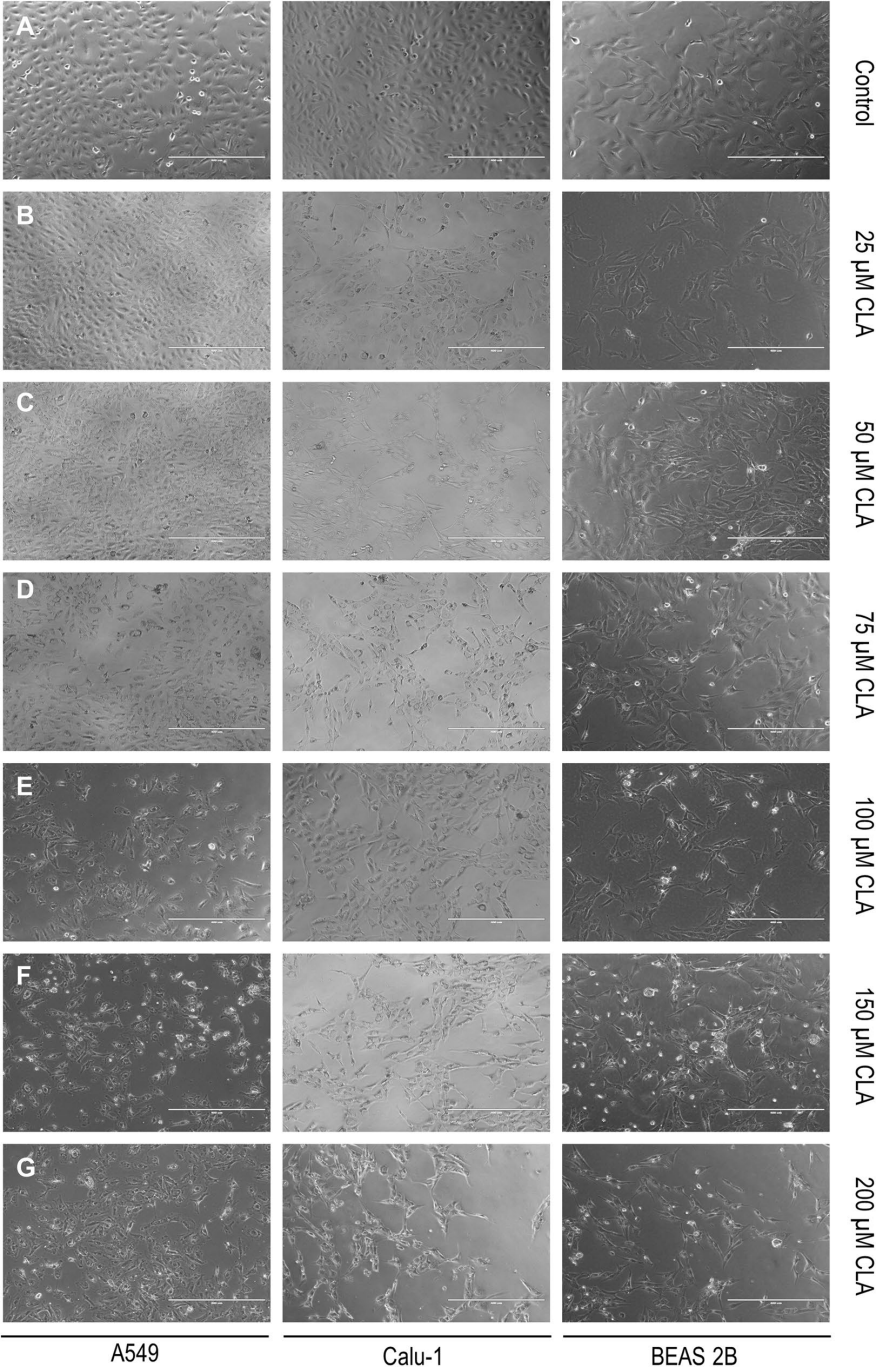
Standard microscopic picture of A549, Calu-1 and Beas-2B cell after CLA treatment—**a** control (CLA-untreated, DMSO-treated cells); **b** cells treated with 25 μM CLA; **c** cells treated with 50 μM CLA; **d** cells treated with 75 μM CLA; **e** cells treated with 100 μM CLA; **f** Cells treated with 150 μM CLA; **g** Cells treated with 200 μM CLA. Fluorescent signal was detected by fluorescence microscope magnification =  × 40

All results concerning the influence of c9, t11 CLA isomer on cell viability, proliferation and morphology are shown in Figs. 4, 5, 6, 7, 8. In order to examine the effect of c9, t11 CLA on A549, Calu-1 and Beas-2B cell viability and proliferation, we used the MTT assay. As described above, we treated cells with different doses of CLA, over a period of 24, 48 and 72 h. We observed that CLA significantly decreased cell proliferation of every tested cell line, regardless of its concentration (Fig. 4a–c). The strongest effect was noticed after 72 h of incubation, at the highest applied doses. In addition to MTT assay we decided to stain all cells with Hoechst 33,342 and PI dyes in order to determine cell membrane integrity. While Hoechst 33,342 (blue) uptake is observed in every cell, irrespective of cell viability, the PI (red) is meant to stain cells with disturbed membrane permeability, which is known characteristic feature of late-apoptotic or necrotic cells. As presented on Figs. 5, 6, 7, the strongest fluorescence signal of PI was observed in cells treated with the highest doses of the CLA (150; 200 µM). In addition, the CLA-related effect can also be observed in the standard microscopic images, where the number of cells and changes in cell morphology are visible (Fig. 8). The number of cells present in culture vessels decreased with increasing doses of CLA. Moreover, those cells exposed to CLA showed some apoptosis-related morphological changes like nuclei fragmentation (Fig. 6d, e) or presence of small intracellular bodies (Fig. 8d–f) compared to respective controls.

## Discussion

LC development is directly connected to an unhealthy lifestyle. It is commonly known that many people with this disease are current or former smokers [2, 27]. Nevertheless, the growing incidence of LC in never smokers [28], caused mainly by growing air pollution [3], or second-hand smoking forces science to seek additional preventive and prophylactic factors. It is well known that cancer cells are characterized by an impaired balance between death and proliferation. A deep understanding of this disparity should improve and speed up the discovery of improved preventive agents. CLA seems to be one such factor. This fatty acid naturally occurs as a mixture of isomers, where the c9,t11 isomer is the most abundant. CLA is predominantly present in ruminant meat and dairy products and is currently a popular compound in various dietary supplements [4, 6]. CLA was first discovered in 1987 by Pariza et al. [6]. Since then, numerous studies have been carried out, pinpointing the anticancer potential of CLA [29], especially in colon, stomach, prostate and hepatic cancer [8-10, 30, 31]. One of the target proteins through which CLA can interact is PPAR-γ [13], the activation of which is related to pro-apoptotic and antiproliferative features [19, 21].

Even though much is known about PPAR-γ activation by its ligands, there are only a few reports considering their effect on *PPARG* expression. The regulation mechanism of *PPARG* transcription has been extensively studied and can be associated with numerous factors. However, the vast majority of available data focus on the regulation of *PPARG* expression in adipose tissue (where it acts as a crucial regulator of adipocytes differentiation), and this mechanism in other cells still remains elusive [32, 33]. The previous research presented by Lu et al., focused on hepatocellular carcinoma, motivated us to investigate the CLA potential to upregulate *PPARG* expression level in LC [8]. In our present study, we focused on the impact of c9,t11 CLA, which is an activator of PPAR-γ protein, on PPAR-γ transcript and protein levels in NSCLC cells and in normal human bronchial epithelial cell line. Similar to Lu et al. results we showed that in all investigated cell types, c9,t11 CLA was able to significantly elevate PPAR-γ mRNA and protein levels. This effect was most noticeable in Beas-2B and A549 cells. We did not find any previous research that describes a similar phenomenon in normal lung cells or NSCLC cell lines. However, aforementioned study performed by Lu et al. demonstrated that both PPAR-γ transcript and protein levels could be enhanced by c9,t11 CLA in human hepatocellular carcinoma cell lines [8]. In addition, research presented by Ramiah et al. showed that *PPARG* mRNA level can be upregulated in the liver of chickens fed by CLA [34]. Together, these results confirm that this particular isomer of linoleic acid is not only an activator of PPAR-γ protein but can also act as a positive regulator of its expression.

It is also worth emphasizing that the application of c9,t11 CLA, and the subsequent increase in PPAR-γ in Lu's et al. investigation, were associated with a strong apoptotic response mediated by changes in the level of apoptosis-related proteins, mainly by upregulation of BAX and/or downregulation of BCL-2 at both, mRNA and protein levels, which resulted in disturbances in the BAX/BCL-2 ratio [8]. This anticancer effect triggered by CLA is corroborated by ample research [7, 9, 10, 29. The Activation of PPARγ can induce apoptosis in several different ways which are thoroughly described by Elrod et al. [18]. In one of the signalling pathways, PPAR-γ is able to bind to the nuclear NF-κB factor site, located in the promoter sequences of *TP53*, thus enhancing its expression [35]. Additionally, it has been shown, that some PPARγ ligands (especially thiazolidinediones) enhance the recruitment of PPARγ to the *TP53* promoter [36]. Consequently, the p53 protein is responsible for the transcriptional activation of several apoptosis-related genes i.e. *BAX* or *DR5* [37].

In our work we obtained a cellular response leading to an upregulation of *PPARG* in all investigated cell lines, but *BAX* expression was only induced in normal bronchial epithelial cells. Surprisingly, we noticed an elevation of anti-apoptotic *BCL-2* gene expression at both mRNA and protein level in investigated NSCLC cells. However, the results of MTT assay depicted that CLA is an effective compound that inhibits NSCLC cells proliferation. The higher doses and/or longer incubation periods with CLA, the more prominent growth inhibition of treated cells was observed, compared to the respective controls. The fluorescent staining confirmed, that c9, t11 CLA isomer can cause cell death and lead to limited cell proliferation. After CLA treatment we observed differences of cell morphology between exposed and control cells. We also detected the PI-derived red fluorescent signal emitted by cells incubated with high doses of mentioned compound, which is an evidence of disturbed membrane permeability. These results, together with an increased expression of *PPARG* may suggest that CLA can cause cells death trough PPAR-γ dependant mechanism. However, we cannot clarify whether this process is associated with the BAX pathway, as in our experiments we did not observe an upregulation of *BAX* mRNA level. Conversely, we detected an increased expression of *BCL-2*. Similar results were presented in the research performed by Majumder and co-workers. They investigated the influence of CLA on key apoptotic genes in human breast cancer cells and noticed that *BCL-2* transcripts were significantly elevated in MDA-MB-231 cells after CLA stimulation. Nevertheless, in those cells CLA was also able to elevate the amount of pro-apoptotic BAX and BCL-XS proteins, which resulted in a higher ratio of these proteins to BCL-2 and promoted cell apoptosis [38]. Unfortunately, the content of BAX protein in investigated NSCLC cells was not assessed in our work, which leaves the open door for further research. Moreover, there are studies showing that, PPAR-γ and p53 proteins are involved in triggering cell apoptosis trough other mechanisms, like the DNA fragmentation and caspase-9 pathway [39]

In recent studies, it has also been shown that the increased quantity of ligand-activated PPAR-γ can act as a protective, anti-apoptotic factor by an upregulation of the BCL-2 protein level [22–24, 40, 41]. This effect was achieved through the application of thiazolidinediones [40] (especially rosiglitazone [22, 23, 41]) and prostaglandins [42], which are also strong PPAR-γ agonists. The authors emphasize the crucial role of PPAR-γ in the cellular defence against oxidative stress, ischaemia or stroke, especially in neural and cardiac cells. Nevertheless, Fong et al. indicated that the mechanism of this effect remains elusive. These findings suggest that cellular effects exerted by PPAR-γ may be different in various types of cells. It is important to mention that the concentration of stimulant may be not without significance concerning the pro- or anti-apoptotic role of PPAR-γ [24].

Another interesting report draws attention to the PPAR-γ status in primary NSCLC tissues, highlighting that the expression level of *PPARG* in lung tumours is usually considerably higher in comparison to corresponding histopathologically unchanged samples, however, this intratumoural *PPARG* overexpression does not exhibit an anticarcinogenic/protective effect because of the lack of PPAR-γ agonists [43, 44]. Li and co-workers proved that the addition of PPAR-γ activating ligand (troglitazone) lead to an inhibition of lung cancer cells proliferation, previously induced by carcinogenic chemicals of cigarette smoke [44]. In the light of these results, CLA appears to be a potent anticancer agent, as it not only elevates the expression of PPAR-γ, but it also acts as its ligand, contributing to decreased NSCLC cells proliferation. In our study, we also analysed the changes in the expression of the *CDKN1A* gene, encoding a cyclin-dependent kinase inhibitor, p21, which is a key factor participating in cell cycle control and arrest. Previous studies reported the inducing effect of PPAR-γ activators on p21 expression in colon and lung cancer cell lines [45–48]. It was shown that the PPAR-γ ligands (PGJ2 and ciglitazone) inhibited the growth and induced apoptosis of several lung carcinoma cell lines. Those processes were mediated by an induction of *CDKN1A* gene expression [46]. In addition, the progression of colon cancer cells was also inhibited after treatment with physiological concentrations of CLA, which was connected with an upregulation of p21 [45]. Although, we clearly demonstrated that CLA induces the mRNA and protein content of PPAR-γ in A549, Calu-1 and Beas-2B cells, we did not detect any significant changes concerning *CDKN1A* transcript levels. A small upregulation of *CDKN1A* expression occurred only in Calu-1 cell line at the dose of 75 µM of CLA. It is worth to mention that the transcriptional regulation of *CDKN1A* is a complex process. Han et al. showed that in case of PPAR-γ ligands such as PGJ2 and ciglitazone there was an enhanced binding activity of Sp1 and C/EBP transcription factors sites in the promoter region of *CDKN1A*, which led to its overexpression [46]. On the other hand, there are also works that pinpointed that the expression of some genes is diminished by PPAR-γ ligands, as in this situation they negatively regulate the binding activity of the same transcription factors (Sp1 and C/EBP) [49, 50].Thus, the influence of PPAR-γ ligands concerning gene expression issue may be cell type specific. On the other hand, Miller et al. demonstrated that the induction of p21 is rapid and does not last long. In their study, the strongest increase in p21 protein and transcript levels was observed after 6 h of incubation with PPAR-γ activator; however, after 24 h, this effect diminished [51]. As our shortest incubation period with CLA was 24 h, it is possible that we could omit any possible changes in *CDKN1A* mRNA level. In conclusion, we showed for the first time that c9,t11 CLA can significantly induce *PPARG* expression in NSCLC and in normal human bronchial epithelial cells. We also showed that the inhibition of NSCLC cell proliferation mediated by CLA may be connected with its stimulatory effect triggered on PPAR-γ. However, there are many pathways activated by PPAR-γ that can lead to the cell growth arrest and apoptosis. Moreover, the apoptotic response can differ depending on various factors, i.e. type of tissue or cell line, or the type and concentration of PPAR-γ activator. We were able to demonstrate that apart from the induction of *PPARG* expression in investigated cell lines, CLA definitely exerts antiproliferative effect and causes prompt morphological changes in our cells (cytoplasmic vacuolization and appearance of enlarged cells with micronuclei). Thus, our study supports the proven antiproliferative and anticancer potential of CLA. However, we want to point out that the effect exerted by the CLA also concerned the normal human bronchial epithelial cells (Beas-2B), which is worth considering in terms of the safety of using this compound. Is CLA a noteworthy prophylactic or protective agent? Ample research supports this idea, and we are not going to question it. What is more important, the precise mechanism that lead to an inhibition of cell proliferation after CLA treatment, alongside *PPARG* overexpression in NSCLC cells need to be further clarified.
